# Regional contributions to left ventricular stroke volume determined by cardiac magnetic resonance imaging in cardiac resynchronization therapy

**DOI:** 10.1186/s12872-021-02325-7

**Published:** 2021-10-26

**Authors:** Björn Östenson, Ellen Ostenfeld, Anna Werther-Evaldsson, Anders Roijer, Zoltan Bakos, Mikael Kanski, Einar Heiberg, Håkan Arheden, Rasmus Borgquist, Marcus Carlsson

**Affiliations:** 1 Clinical Physiology, Department of Clinical Sciences Lund, Lund University, Skåne University Hospital, Lund, Sweden; 2 Section for Heart Failure and Valvular Disease, Department of Clinical Sciences Lund, Cardiology, Lund University, Skåne University Hospital, Lund, Sweden; 3 Section of Arrhythmia, Department of Clinical Sciences Lund, Cardiology, Lund University, Skåne University Hospital, Lund, Sweden; 4 Department of Radiology, New York University School of Medicine, New York, NY USA; 5 Wallenberg Center for Molecular Medicine, Lund University, Lund, Sweden

**Keywords:** Cardiac resynchronization therapy, Cardiac magnetic resonance, Predictors

## Abstract

**Background:**

Cardiac resynchronization therapy (CRT) restores ventricular synchrony and induces left ventricular (LV) reverse remodeling in patients with heart failure (HF) and dyssynchrony. However, 30% of treated patients are non-responders despite all efforts. Cardiac magnetic resonance imaging (CMR) can be used to quantify regional contributions to stroke volume (SV) as potential CRT predictors. The aim of this study was to determine if LV longitudinal (SV_long%_), lateral (SV_lat%_), and septal (SV_sept%_) contributions to SV differ from healthy controls and investigate if these parameters can predict CRT response.

**Methods:**

Sixty-five patients (19 women, 67 ± 9 years) with symptomatic HF (LVEF ≤ 35%) and broadened QRS (≥ 120 ms) underwent CMR. SV_long%_ was calculated as the volume encompassed by the atrioventricular plane displacement (AVPD) from end diastole (ED) to end systole (ES) divided by total SV. SV_lat%_, and SV_sept%_ were calculated as the volume encompassed by radial contraction from ED to ES. Twenty age- and sex-matched healthy volunteers were used as controls. The regional measures were compared to outcome response defined as ≥ 15% decrease in echocardiographic LV end-systolic volume (LVESV) from pre- to 6-months post CRT (delta, Δ).

**Results:**

AVPD and SV_long%_ were lower in patients compared to controls (8.3 ± 3.2 mm vs 15.3 ± 1.6 mm, *P* < 0.001; and 53 ± 18% vs 64 ± 8%, *P* < 0.01). SV_sept%_ was lower (0 ± 15% vs 10 ± 4%, *P* < 0.01) with a higher SV_lat%_ in the patient group (42 ± 16% vs 29 ± 7%, *P* < 0.01). There were no differences between responders and non-responders in neither SV_long%_ (*P* = 0.87), SV_lat%_ (*P* = 0.09), nor SV_sept%_ (*P* = 0.65). In patients with septal net motion towards the right ventricle (n = 28) ΔLVESV was − 18 ± 22% and with septal net motion towards the LV (n = 37) ΔLVESV was − 19 ± 23% (*P* = 0.96).

**Conclusions:**

Longitudinal function, expressed as AVPD and longitudinal contribution to SV, is decreased in patients with HF scheduled for CRT. A larger lateral contribution to SV compensates for the abnormal septal systolic net movement. However, LV reverse remodeling could not be predicted by these regional contributors to SV.

## Background

Cardiac dyssynchrony in chronic heart failure (HF) may be restored by cardiac resynchronization therapy (CRT), which induces coordinated contraction in the left ventricle [1]. The current indications for CRT from European Society of Cardiology (ESC) are symptomatic HF despite optimal medical treatment, left ventricular ejection fraction (LVEF) ≤ 35% and broadened QRS complex (≥ 130 ms), preferably with left bundle branch block (LBBB) [2]. In large randomized controlled trials, CRT has been shown to prolong survival, stall progression, and reduce symptoms of HF [3]. However, approximately 1/3 of patients undergoing CRT treatment are objective non-responders [3]. Consequently, there is a need for reliable preoperative predictive factors of response to CRT. Cardiac magnetic resonance imaging (CMR) is gold standard for cardiac volumes and infarct detection. However, neither randomized CMR trials nor more advanced echocardiographic markers have overall not shown a benefit of using imaging beyond LVEF in the selection of CRT candidates [4, 5].

Conflicting results have been presented regarding the ability of multi-modality imaging to guide CRT-lead placement. Sommer et al. showed in a single-center randomized controlled trial that a multi-modality imaging approach improved the CRT effect [6]. However, the randomized clinical trial CRT CLINIC study could not show any benefit of assessing the most suitable segments prior to lead placement by using the combination of two-dimensional speckle tracking echocardiography (latest segment activation), cardiac CT (suitable cardiac vein anatomy), and CMR (scar evaluation) [7]. Thus, further studies are needed to identify imaging biomarkers that can help select patients more likely to respond to CRT treatment.

CMR can quantify the stroke volume (SV) generated from LV septal net movement in addition to the longitudinal and lateral contributions to SV [8, 9]. In healthy subjects, the septum contributes to about 7% of LVSV [10]. However, this may differ in patients with increased QRS duration, where abnormal septal motion often is present [11]. Thus, septal contribution to LVSV may be a prognostic factor of CRT response.

The longitudinal component of LV stroke volume (SV_long%_) is generated by the atrioventricular plane displacement (AVPD). Impaired longitudinal ventricular function has been shown to be associated with adverse events, even in the absence of reduced LVEF [12]. However, the possible prognostic value of longitudinal function for predicting treatment response has not been studied in a CRT population.

While other image predictors for response to CRT have been studied, for example apical rocking [13] and myocardial strain [14] with echocardiography, regional contributions to SV with CMR have not been studied in CRT patients. Regional contributions to SV provide, in contrast to other imaging indicators of dyssynchrony, quantitative measurements of how much each of the longitudinal, lateral, and septal displacements generate SV. These three imaging predictors could potentially be used as reliable imaging predictors of response to CRT before implantation, reducing the number of non-responders. Our hypothesis was that measures of regional contributions to SV could be used to select appropriate patients for CRT.

Therefore, the aims of this study were to (1) determine if longitudinal, lateral, and septal contributions to SV in patients with HF and eligible for CRT differ from healthy controls, (2) investigate if these parameters predict CRT-treatment outcome defined as reverse remodeling at 6 months after CRT implantation, and (3) investigate if there were differences in regional contribution to SV in patients with ischemic versus non-ischemic etiology.

## Methods

### Study population

**Fig. 1:**
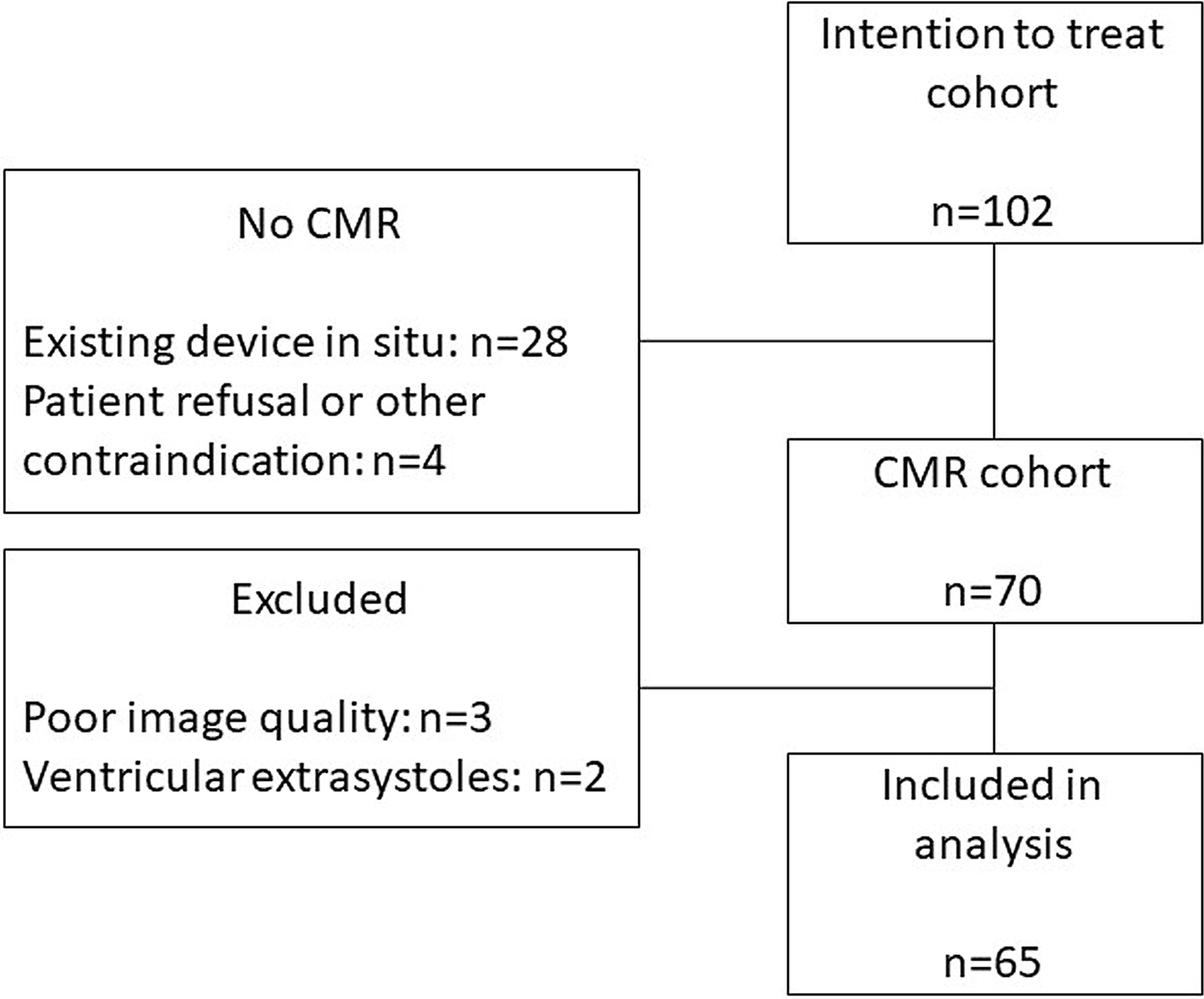
Flow chart of study participants. *CMR* cardiac magnetic resonance imaging

This is a sub-study of a randomized clinical trial, which prospectively included patients with indication for CRT between 2011 to 2017 at Skåne University Hospital, Sweden (ClinicalTrials.gov: NCT01426321). Indication for CRT was New York Heart Association (NYHA) class II-IV despite optimal medical therapy, QRS-complex ≥ 120 ms on standard electrocardiogram (ECG), and LVEF ≤ 35% [15]. Patients with a life expectancy < 12 months, myocardial infarction within 3 months of enrolment, moderate-severe valve disease, chronic atrial fibrillation, and severely reduced renal function (estimated glomerular filtration rate < 30 ml/min) were excluded from the study. The results of the randomized trial were neutral with respect to response to therapy between the two randomized groups, LVESV increase ≥ 15% at six months in 56% of patients in the intervention group versus 55% in controls, *P* = 0.96 [15]. Therefore, patients were pooled in this exploratory sub-study. Of a total of 102 patients in the main study, 28 already had devices in situ, and 4 were excluded due to patient refusal or other contraindication, leading to 70 patients undergoing CMR examination. Five of these patients were excluded owing to poor image quality (n = 3) or arrhythmia during examinations (n = 2). Hence, 65 patients were analyzed (Fig. 1).

Ischemic heart disease was defined as fulfillment of at least one of the following criteria: (1) medical history of myocardial infarction or revascularization (percutaneous coronary intervention or coronary artery bypass grafting), (2) lumen stenosis ≥ 50% in left main coronary artery or proximal left anterior descending artery, and (3) lumen stenosis ≥ 75% in any epicardial vessel. Non-ischemic heart disease was defined as patients not fulfilling any of these criteria [16].

As control group, 20 healthy age- and sex-matched individuals (8 women, 62 ± 11 years) with normal blood pressure (< 140/90 mmHg), no past cardiovascular diseases, no cardiovascular treatments, and with normal ECG findings were recruited.

### Cardiac magnetic resonance imaging

All subjects underwent CMR examination at baseline using a Philips Intera 1.5 T or 3.0 T (Philips Medical System, Best, The Netherlands), or Siemens Aera 1.5 T (Siemens Medical Systems, Erlanger, Germany). Balanced steady-state free precession cine images were collected in the supine position during end-expiratory apnea and included a short-axis cine stack, and long-axis cines in 2-chamber, 3-chamber, and 4-chamber views. CMR was not performed at six months follow-up due to CRT devices were not MRI compatible.

Retrospective ECG triggering was used to acquire images with reconstructed temporal resolution of 30 time frames per cardiac cycle. Typical imaging parameters were TR/TE 3 ms/1.3–1.8 ms, flip angle 45° (3 T) or 60° (1.5 T), and slice thickness 6–10 mm with 0–2 mm slice gap.

In patients, late gadolinium (Gd) enhancement (LGE) images were acquired to quantify the extent of myocardial fibrosis. Intravenous Gd-based contrast of 0.2 mmol/kg (gadoteric acid, Gd-DOTA, Guerbet, Gothia Medical AB, Billdal, Sweden) was injected in an antecubital vein after which inversion-recovery or phase sensitive inversion recovery sequences were used to generate LGE images. Typical image parameters were TR/TE 4.2–5.2 ms/1.0–3.3 ms, flip angle 15° (1.5 T) or 25° (3 T), and slice thickness 6–10 mm with 0–2 mm slice gap.

### Image analysis

CMR image analysis was performed using the software Segment v2.0 R5039 (Medviso, Lund, Sweden: http://segment.heiberg.se) [17]. Short-axis images were delineated according to current consensus document of standardized image interpretation, including trabeculations and papillary muscles in the cavity volumes [18]. LV epicardial and endocardial borders were delineated at end diastole (ED) and end systole (ES) for measurements of ED volume (EDV), ES volume (ESV), and LV mass. SV was defined as EDV-ESV. LVEF was defined as SV/EDV. LV mass was calculated by multiplying the myocardial volume with the muscle density (1.05 g/ml). Fibrosis was visually determined as present/absent by an experienced physician (> 20 years of experience).

### Longitudinal contribution to stroke volume

**Fig. 2:**
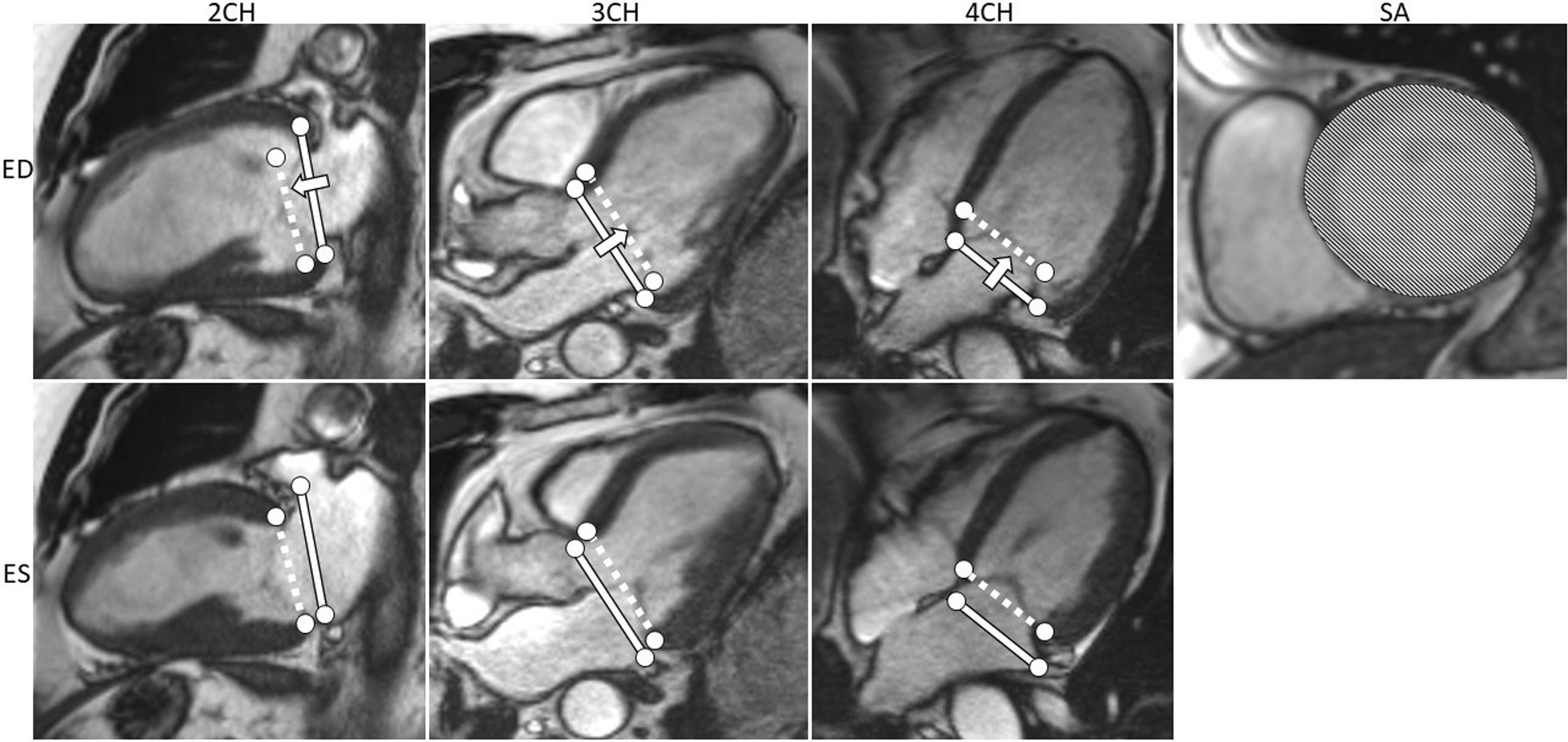
Atrioventricular plane displacement and longitudinal contribution to stroke volume (SV_long%_). CMR images in 2-chamber (2CH), 3-chamber (3CH), 4-chamber (4CH), and short-axis (SA) views at end diastole (ED, upper panes) and end systole (ES, lower panes). The solid lines indicate the atrioventricular planes at ED and the dashed lines at ES. The atrioventricular plane displacement (AVPD) is defined as the distance the atrioventricular plane moves from ED to ES in the indicated direction (arrow). The SV_long%_ is calculated by multiplying the AVPD with the epicardial short-axis area in ED (marked area in the SA view at the level of the dashed lines) comprising the atrioventricular plane motion, divided with the left ventricular stroke volume

The longitudinal contribution to SV (SV_long%_) was measured as previously described [8, 9, 19]. In short, AVPD is defined as the displacement of the atrioventricular plane between ED and ES (Fig. 2). Six standardized landmarks were used to measure the LV atrioventricular plane: from the 4-chamber view: LV inferoseptal and LV anterolateral; from the 3-chamber view: LV anteroseptal and LV inferolateral; and from the 2-chamber view: LV anterior and inferior. The short-axis area comprising the range of the AVPD was calculated using the epicardial delineation in the short-axis views, as previously discussed [9]. The average of the two largest epicardial short-axis areas encompassed by the AVPD motion was multiplied with the AVPD to calculate the absolute longitudinal contribution to SV in ml. The longitudinal contribution relative to total SV (SV_long%_) was calculated by dividing the absolute longitudinal contribution to SV (in ml) with the total LVSV.

### Radial contribution to stroke volume

**Fig. 3:**
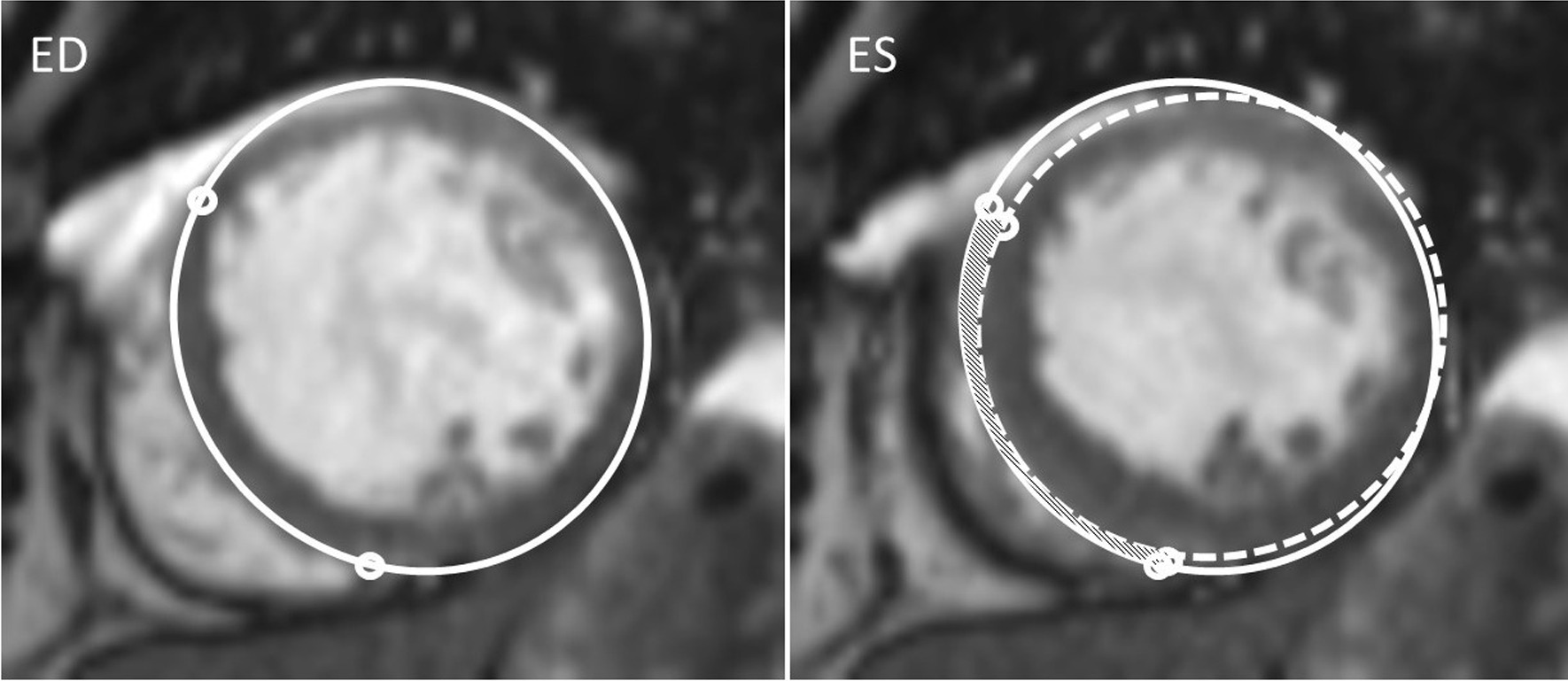
Septal and lateral contribution to stroke volume (SV_sept%_ and SV_lat%_). Short-axis views in end-diastole (ED, left pane) and end-systole (ES, right pane). Left ventricular (LV) epicardial border in ED is represented by the solid line. LV epicardial border in ES is represented by the dashed line. Right ventricular (RV) insertion points are represented as white circles. SV_sept%_ is measured with the area defined by the RV insertion points and the septal borders in ED and ES (marked area). SV_lat%_ is measured with the area defined by the lateral LV borders between ED and ES, excluding the septum

The radial contribution to SV was measured as previously described using an in-house developed plugin to the Segment software [20]. In short, the septal contribution to SV (SV_sept%_) was defined by the area enclosed by the septal epicardial contours and the RV insertion points between ED and ES in the short-axis images (Fig. 3). The lateral contribution to SV (SV_lat%_) was defined by the area enclosed by the lateral LV epicardial contours and the RV insertion points in ED and ES in the short-axis images (Fig. 3).

### Echocardiography

Echocardiography was acquired with a Vivid E9 (GE Medical, Horten, Norway) using an M5Sc-D probe with standard projections according to guidelines [21]. Echocardiographic assessment of LV volumes was analyzed in EchoPac BT12 (GE Medical, Horten, Norway) offline. Patients were examined prior to and six months after CRT implantation in accordance with study protocol [8].

The effect of CRT was evaluated with echocardiography at baseline prior to CRT implantation and at six months follow-up after implantation to assess reverse remodeling. Response to CRT was defined as previously proposed [22] as ≥ 15% reduction in LVESV measured by the Simpson’s biplane method [23]. Dyssynchrony was assessed at baseline and was defined as anteroseptal to posterior mid-left ventricular delay ≥ 130 ms using radial speckle-tracking analysis as previously described [7].

### Statistical analysis

Statistical analyses were performed using IBM SPSS version 25 (SPSS inc., Chicago, Illinois, USA).

Continuous variables are expressed as mean ± SD. Normal distribution was determined by visual assessment of histograms. For SV_sept%_, the cutoff value was 0%, with negative contribution to LVSV as a sign of cardiac dyssynchrony [24]. Groups were compared with unpaired student’s t-tests for continuous variables and Pearson’s chi-squared test or Fisher’s exact test for categorical variables. Individual baseline and follow-up data were compared using paired student’s t-tests. Results with *P* values < 0.05 were considered statistically significant. Univariable and multivariable logistic regression analyses were conducted with responder as dependent variable and SV_long%_, SV_lat%_, and SV_sept%_ as predictors. Demographic, clinical, and echocardiographic predictors were added to the model to adjust for confounding factors. Candidate predictors in the univariable logistic analysis that had a *P* value of < 0.25 were included in the multivariable analysis [25]. Included predictors were retained in the model if *P* < 0.05. Post-hoc sample size analysis was conducted using the software PS: Power and Sample Size Calculation (version 3.1.6, October 2018) [26]. Assumptions included alpha level of 0.05, statistical power of 0.8, and effect size and standard deviations from the present study.

## Results

### Study population

Sixty-five patients fulfilled the study protocol. Nineteen were women, mean age was 67 ± 9 years. 51% had non-ischemic etiology and QRS duration on standard ECG was 167 ± 17 ms. Mean ejection fraction was 26 ± 8%, and 86% had typical LBBB. The characteristics of the cohort are in detail presented in Table 1.

**Table 1:** Baseline patient characteristics

	Patients (n = 65)	Controls (n = 20)
Women	19 (29%)	8 (40.0%)
Age (years)	67 ± 9^†^	62 ± 11
BSA (m^2^)	2.0 ± 0.2^†^	1.9 ± 0.2
Diabetes	9 (14%)	0
QRS-duration (ms)	167 ± 17^†††^	95 ± 11
LBBB	56 (86%)	N/A
Heart rate (beats/min)	67 ± 13^†^	62 ± 7
NIBP (mmHg)		
Systolic	127 ± 17	131 ± 13
Diastolic	76 ± 9	75 ± 7
Medication		0
ACEI/ARB	65 (100%)	
Betablocker	58 (89%)	
Diuretics	56 (86%)	
Antihyperlipidemics	41 (63%)	
Platelet aggregation inhibitors	35 (54%)	
NYHA Class		N/A
NYHA Class II	20 (31%)	
NYHA Class III	41 (63%)	
NYHA Class IV	4 (6%)	
Etiology		
Ischemic	32 (49%)	
Non-ischemic	33 (51%)	
LVEDV (ml)	326 ± 115^†††^	163 ± 37
LVESV (ml)	246 ± 110^†††^	66 ± 21
LVSV (ml)	80 ± 23^††^	97 ± 20
LVEF (%)	26 ± 8^†††^	60 ± 5
LV mass (g)	170 ± 49^†††^	112 ± 32
CO (l/min)	5.3 ± 1.5^†^	6.0 ± 1.2
LVAVPD (mm)	8.3 ± 3.2^†††^	15.3 ± 1.6
SA area (cm^2^)	51 ± 11^†††^	34 ± 7
SV_long%_ (%)	53 ± 18^††^	64 ± 8
SV_lat%_ (%)	41 ± 16^†††^	29 ± 7
SV_sept%_ (%)	0 ± 15^†††^	10 ± 4
LGE positive	40 (62%)	N/A

Continuous variables are expressed as means ± SD and categorical values in absolute numbers and proportion in parenthesis

ACEI, angiotensin-converting enzyme inhibitor; ARB, angiotensin II receptor blocker; BSA, body surface area; CO, cardiac output; DCM, dilated cardiomyopathy; IHD, ischemic heart disease; LBBB, left bundle branch block; LV, left ventricular; AVPD, atrioventricular plane displacement; EDV, left ventricular end-diastolic volume; EF, left ventricular ejection fraction; ESV, left ventricular end-systolic volume; SV, left ventricular stroke volume; NCC, non-compaction cardiomyopathy; NIBP, non-invasive systemic blood pressure; NYHA, New York Heart Association; SV_lat%_, lateral contribution to stroke volume; SV_long%_, longitudinal contribution to stroke volume; SV_sept%,_ septal contribution to stroke volume

^†^*P* < 0.05 patients vs healthy controls

^††^*P* < 0.01 patients vs healthy controls

^†††^*P* < 0.001 patients vs healthy controls

### Regional contributions in CRT candidates and healthy controls

In patients receiving CRT, the relative regional contributions to LVSV were for SV_long%_ 53 ± 18%, for SV_lat%_ 41 ± 16%, and for SV_sept%_ 0 ± 15%. Negative (abnormal) SV_sept%_ was found in 43% of the patients eligible for CRT. Thus, just above half of the selected CRT candidates did not have a septal net movement towards the right ventricle during systole.

Compared to controls, the longitudinal shortening expressed as AVPD (8.3 ± 3.2 vs 15.3 ± 1.6 mm, *P* < 0.001) and SV_long%_ were lower in patients (53 ± 18 vs 64 ± 8%, *P* < 0.01). Also, patients had a lower SV_sept%_ (0 ± 15% vs 10 ± 4%, *P* < 0.001) accompanied with an increase in SV_lat%_ (41 ± 16% vs 29 ± 7%, *P* < 0.001). Absolute SV was lower (80 ± 23 ml vs 97 ± 20 ml, *P* < 0.01) and the short-axis area was larger in patients compared to controls (51 ± 11 cm^2^ vs 34 ± 7 cm^2^, *P* < 0.001) (Table 1).

### Outcome

**Fig. 4:**
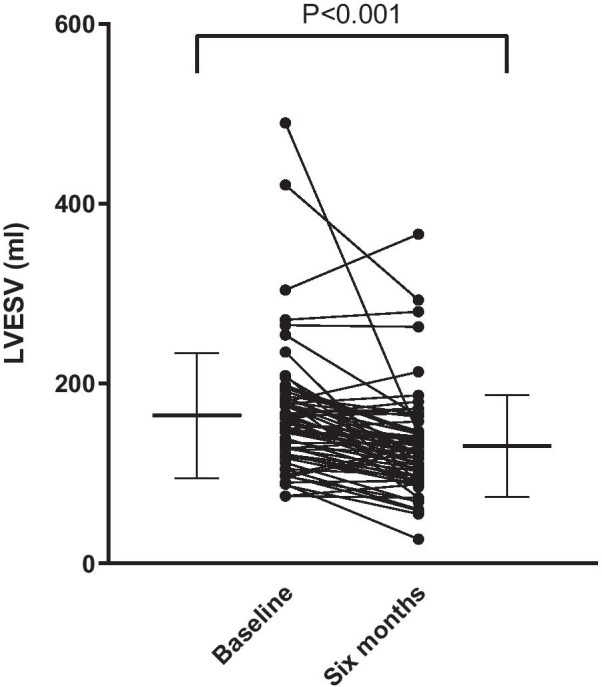
Illustration of reverse remodeling. Paired individual values at baseline and six months follow-up. Error bars denote mean values ± SD. There was a significant reduction in left ventricular end-systolic volume (LVESV) by echocardiography six months after CRT implantation

At six months follow-up echocardiography, LVESV was decreased ≥ 15% in 37 patients (57%), as an objective marker of reverse remodeling and a positive response to CRT. The mean ΔLVESV was − 18 ± 22% (*P* < 0.001, Fig. 4). Tables 2 and 3 show the ability of each individual proposed CMR predictor to stratify patients according to reverse remodeling response. There were no differences in SV_long%_, SV_lat%_, nor SV_sept%_ between CRT responders and non-responders (Table 3).

**Table 2:** Comparison of patients with negative and positive septal contribution to stroke volume

CMR parameter	ΔLVESV ≥ 15% (n = 37)	ΔLVESV < 15% (n = 28)	*P*	ΔLVESV (%)	*P*
Negative SV_sept%_	16	12	0.98	− 18 ± 22	0.96
Positive SV_sept%_	21	16		− 19 ± 23	

Cutoff for SV_sept%_ was 0%. Continuous variables are expressed as means ± SD. No regional contribution parameter was associated with ≥ 15% reduction in left ventricular end-systolic volume (ΔLVESV) on echocardiography after six months follow-up

**Table 3:** Comparison of regional contributions to SV in responders and non-responders to CRT

	Responders ΔLVESV ≥ 15% (n = 37)	Non-responders ΔLVESV < 15% (n = 28)	*P*
LVAVPD (mm)	8.0 ± 3.1	8.8 ± 3.2	0.31
SV_long%_ (%)	53 ± 18	53 ± 19	0.87
SV_lat%_ (%)	38 ± 15	45 ± 16	0.09
SV_sept%_ (%)	− 1 ± 17	1 ± 11	0.65
LGE positive	23 (62%)	17 (61%)	0.91
Non-ischemic/ischemic heart disease	23 (62%)/14 (38%)	10 (36%)/18 (64%)	< 0.05
Anterioseptal to posterior mid-left ventricular delay ≥ 130 ms	15 (41%)	12 (46%)	0.66
QRS duration ≥ 150 ms	34 (92%)	23 (82%)	0.28
LBBB	34 (92%)	22 (79%)	0.16
Male/female sex	27 (73%)/10 (27%)	19 (68%)/9 (32%)	0.65

Responders vs non-responders. Response is defined as a negative change in left-ventricular end-systolic volume (ΔLVESV) of ≥ 15% by echocardiography. Values are expressed as means ± SD or absolute number and proportion in parenthesis

CRT: cardiac resynchronization therapy; LBBB: left bundle branch block; LVAVPD: left ventricular atrioventricular plane displacement; SV_long%_: longitudinal contribution to SV; SV_lat%_: lateral contribution to stroke volume; SV_sept%_: septal contribution to stroke volume

Forty-six patients (71%) improved in NYHA functional classification ≥ 1 at six months follow-up. Twenty-seven out of 63 patients (43%) had dyssynchrony on echocardiography at baseline. Two patients were not assessable for dyssynchrony analysis. There were no associations between dyssynchrony and CRT outcomes, defined as LVESV reduction ≥ 15% (*P* = 0.66) and NYHA functional classification improved ≥ 1 (*P* = 0.47).

Multivariable logistic regression to study the effect of SV_long%_, SV_lat%_, and SV_sept%_ on LVESV response was not significant (*P* = 0.17). When demographic, clinical, and echocardiographic variables were added to account for confounding to the model, the model remained non-significant (*P* = 0.10, Table 4). Further, SV_long%_, SV_lat%_, and SV_sept%_ did not predict outcome defined as NYHA response (defined as improvement in ≥ 1 NYHA classification at six months follow-up, Table 5).

**Table 4:** Logistic regression analysis with LVESV response as dependent variable

Dependent variable: LVESV response	Univariable			Multivariable		
	OR	95% CI	*P*	OR	95% CI	*P*
Anteroseptal to posterior mid-left ventricular delay ≥ 130 ms	1.26	0.46–3.46	0.66	–	–	–
QRS duration ≥ 150 ms	2.46	0.54–11.33	**0.25**	2.41	0.45–13.01	0.31
LBBB	3.09	0.70–13.66	**0.14**	2.25	0.46–11.01	0.32
Ischemic Heart Disease	0.34	0.12–0.94	**0.04**	0.46	0.16–1.37	0.16
Male sex	1.28	0.44–3.75	0.65	–	–	–
SV_long%_	1.00	0.98–1.03	0.86	–	–	–
SV_lat%_	0.97	0.94–1.01	**0.09**	0.98	0.94–1.01	0.17
SV_sept%_	0.99	0.96–1.03	0.64	–	–	–

Univariable and multivariable logistic regression analyses. Significant P values in the univariable analysis (cut-off < 0.25) were included in the multivariable analysis and are indicated in bold

LBBB, left bundle branch block; LVESV: left ventricular end-systolic volume; SV_long%_: longitudinal contribution to SV; SV_lat%_: lateral contribution to stroke volume; SV_sept%_: septal contribution to stroke volume

**Table 5:** Logistic regression analysis with NYHA response as dependent variable

Dependent variable: NYHA response	Univariable			Multivariable		
	OR	95% CI	*P*	OR	95% CI	*P*
Anteroseptal to posterior mid-left ventricular delay ≥ 130 ms	1.50	0.50–4.50	0.47	–	–	–
QRS duration ≥ 150 ms	1.54	0.33–7.20	0.59	–	–	–
LBBB	1.25	0.28–5.62	0.77	–	–	–
Ischemic Heart Disease	1.5	0.51–4.41	0.46	–	–	–
Male sex	0.82	0.25–2.71	0.74	–	–	–
SV_long%_	0.98	0.95–1.01	0.23	–	–	–
SV_lat%_	1.00	0.97–1.03	0.99	–	–	–
SV_sept%_	1.00	0.96–1.03	0.79	–	–	–

Univariable and multivariable logistic regression analyses. Significant P values in the univariable analysis (cut-off < 0.25) were included in the multivariable analysis

LBBB, left bundle branch block; NYHA: New York Heart Association; SV_long%_: longitudinal contribution to SV; SV_lat%_: lateral contribution to stroke volume; SV_sept%_: septal contribution to stroke volume

### Ischemic versus non-ischemic

Patients with ischemic and non-ischemic etiology of heart failure were compared in a sub-group analysis of the patient characteristics (Table 6). Patients with ischemic heart failure were older, had more platelet aggregation inhibitor treatment and more LGE, but did not otherwise differ from the non-ischemic patients. Both groups had similar QRS-duration (167 ± 20 vs 167 ± 13 ms, *P* = 0.96) and proportion of LBBB (81% vs 91%, *P* = 0.26). Out of the 33 patients with non-ischemic etiology of heart failure, there were more responders than non-responders to CRT (n = 23 vs n = 10, *P* < 0.05). There was no difference in reverse remodeling between patients with ischemic heart disease compared to patients with non-ischemic heart disease (ΔLVESV − 14 ± 20 ml vs − 23 ± 24 ml, *P* = 0.12). Longitudinal shortening did not differ in patients with ischemic compared to non-ischemic etiology (AVPD 7.7 ± 3.0 mm vs 8.9 ± 3.2 mm, *P* = 0.15). There were no differences in regional contributions to SV between patients with ischemic vs non-ischemic heart disease (Table 6).

**Table 6:** Subgroup analysis of patients with ischemic and non-ischemic heart disease

	Ischemic heart disease (n = 32)	Non-ischemic heart disease (n = 33)	*P*
Women	6 (19)	13 (39)	0.07
Age (years)	71 ± 8	63 ± 8	< 0.001
BSA (m^2^)	1.97 ± 0.19	2.03 ± 0.24	0.26
Diabetes	4 (13)	5 (15)	1.0
QRS-duration (ms)	167 ± 20	167 ± 13	0.96
LBBB	26 (81)	30 (91)	0.30
Heart rate (beats/min)	68 ± 15	67 ± 10	0.87
NIBP (mmHg)			
Systolic	128 ± 18	127 ± 16	0.75
Diastolic	75 ± 8	77 ± 9	0.47
Medication			
ACEI/ARB	32 (100)	33 (100)	1.0
Betablocker	28 (88)	30 (91)	0.66
Diuretics	27 (84)	29 (88)	0.68
Antihyper-lipidemics	29 (91)	12 (36)	
Platelet aggregation inhibitors	24 (75)	11 (33)	0.001
NYHA Class			0.32
NYHA Class II	8 (25)	12 (36)	
NYHA Class III	23 (72)	18 (55)	
NYHA Class IV	1 (3)	3 (9)	
Echocardiagraphy			
ΔLVESV (%)	− 14 ± 20	− 23 ± 24	0.12
CMR			
EDV (ml)	323 ± 68	329 ± 148	0.82
ESV (ml)	242 ± 61	250 ± 143	0.77
SV (ml)	80 ± 26	79 ± 21	0.80
EF (%)	25 ± 7	27 ± 9	0.40
CO (l/min)	5.3 ± 1.6	5.3 ± 1.5	0.90
LVAVPD (mm)	7.7 ± 3.0	8.9 ± 3.2	0.15
SV_long%_	49 ± 17	57 ± 19	0.10
SV_lat%_	45 ± 18	37 ± 13	0.07
SV_sept%_	− 1 ± 14	2 ± 16	0.41
LGE positive	28 (88%)	12 (36%)	< 0.01

Values are expressed as means ± SD or in absolute number and proportion in parenthesis. ΔLVESV, change in left ventricular end-systolic volume from pre to 6 months post CRT echocardiography

ACEI, angiotensin-converting enzyme inhibitor; ARB, angiotensin II receptor blocker; BSA, body surface area; CO, cardiac output; CMR, cardiac magnetic resonance imaging, EDV, end-diastolic volume; EF, ejection fraction; ESV, end-systolic volume; LBBB, left bundle branch block; LVAVPD, left ventricular atrioventricular plane displacement; NIBP, non-invasive systemic blood pressure; NYHA, New York Heart Association; SV, stroke volume; SVlong%, longitudinal contribution to SV; SVlat%, lateral contribution to stroke volume; SVsept%, septal contribution to stroke volume

### Post-hoc sample size analysis

There were no significant differences in LVAVPD, SV_long%_, SV_lat%_, and SV_sept%_ between responders and non-responders as the 95% confidence intervals for the two groups overlapped for all parameters. This study could have yielded false-negative results (i.e. type II error) due to too few participants. To investigate the risk of type II error, a post-hoc sample size analysis was performed. Sample sizes of n = 342, n = 3975, n = 96 and n = 4362 respectively for LVAVPD, SV_long%_, SV_lat%_, and SV_sept%_ would have been needed to detect a signal.

## Discussion

This study shows that patients eligible for CRT treatment have both lower longitudinal systolic atrioventricular plane displacement and septal contribution to SV, compared to healthy controls. However, patients have higher lateral contribution to SV, which is interpreted as a compensatory mechanism to the decreased septal contribution. While this is the first study showing these differences between patients with HF and electromechanical dyssynchrony and healthy controls, the suggested measures of regional function could not predict CRT outcome in terms of reverse remodeling at six months follow-up. In non-ischemic cardiomyopathy, reverse remodeling was more common compared to ischemic disease. Despite this, no difference in regional contribution to SV could be seen between ischemic and non-ischemic patients. Even when assessing with multivariable analysis of demographic, clinical and CMR characteristics, there was no association with responding on CRT treatment in the CMR parameters. Thus, our study is in line with previous studies that could not show any added value of regional function measures to further decrease the number of non-responders in patients selected for CRT treatment compared to current guidelines [4, 27].

### Search for novel prognostic predictors of response to CRT

As of today, presence of LBBB, non-ischemic etiology, sex, QRS-duration, and sinus rhythm are the only factors that have been found to consistently predict response to CRT in randomized prospective trials [28]. Mechanical measures of dyssynchrony have been identified by echocardiography, for example the retrospective cohort study PREDICT-CRT trial which showed that presence of septal flash and apical rocking on echocardiography were markers of mechanical dyssynchrony [29]. However, randomized trials are warranted before new clinical guidelines of patient selection to CRT can be implemented. Other markers of mechanical and electrical dyssynchrony (e.g. left ventricular pre-ejection period, inter- and intraventricular delay by tissue Doppler imaging, and systolic stretch index) have also been investigated using echocardiography [30, 31], but with limited success. A novel approach with echocardiography is to determine wasted septal work with regional pressure-strain loops [11] and this was shown in retrospective data to predict outcome together with septal flash [32]. Mechanical measures of dyssynchrony have also been described in patients with LBBB using CMR [24]. Further, myocardial scar in the paced segment affect CRT effect negatively [33], and scar has been shown in a retrospective observational study to independently predict clinical events and LV functional improvement [34]. However, prospective randomized controlled trials are needed to validate imaging measures as predictors of CRT.

The present prospective observational study aimed to test if longitudinal and septal movements quantified by CMR can predict response to CRT. Longitudinal function defined as AVPD and SV_long%_ was lower in patients eligible for CRT compared to healthy controls. Even if decreased AVPD carries prognostic information of poor outcome in a multitude of diseases, longitudinal function defined as AVPD and SV_long%_ was not related to treatment response in CRT-eligible patients.

In patients referred for CRT, we found a net inverse motion of the septum and thus negative contribution to SV in almost half the patients. This contrasted to healthy controls who all had a positive contribution to SV. However, there was no difference in reverse remodeling 6 months after treatment between patients with negative vs positive septal contribution to LVSV. One explanation for the heterogenous response to CRT may be that differences in regions affected by disease or presence of LBBB give rise to different patterns of mechanical dyssynchrony [24]. Electrical dyssynchrony is not necessarily accompanied with mechanical dyssynchrony susceptible for CRT [35]. Another possible explanation for the lack of response in patients with negative SV_sept%_ is that the measurement was not time resolved. Abnormal septal contractions happening before and after end-systole might therefore have been missed. Post-systolic contraction/shortening is an inefficient contraction after aortic valve closure and has recently gained attention as a potential diagnostic predictor [36]. It is possible that post-systolic contraction reflects cardiac mechanical dyssynchrony susceptible to CRT.

Guiding of CRT using a multi-modality imaging strategy, with a combination of cardiac venous anatomy using cardiac computer tomography, myocardial perfusion using single-photon emission computed tomography, and deformation using speckle-tracking echocardiography, showed improvement of a composite clinical outcome in a prospective randomized controlled single-center trial [6]. Also, combining dyssynchrony and fibrosis data using CMR in a cohort study of 40 patients showed a sensitivity of 90% and specificity of 58% to identify clinical responders [37]. However, in our prospective randomized CRT study we could not improve the degree of reverse remodeling at 6 months when guiding electrode placements using a multi-modality approach of strain data from echocardiography, venography from computer tomography, and fibrosis from CMR [15]. The neutral results from the main study allowed us to perform this sub-study without taking into account if patients were randomized to image-guided electrode placement or not.

### Defining response to CRT

The response rate to CRT is dependent on the selected outcome measure and study population characteristics [38]. In this study, we chose ΔLVESV from echocardiography at six months follow-up as a measure to define reverse remodeling as the primary outcome. This is a surrogate marker that has been shown to be reliable for CRT response [39]. The reverse remodeling response with reduced ΔLVESV to CRT was in accordance to previous studies [40, 41], both regarding mean ΔLVESV and proportion of responders defined as ≥ 15% LVESV reduction after six months. Alternative outcome variables to physiological measures commonly used to evaluate HF progression include clinical assessment, major adverse cardiac events, hospitalization and mortality [42]. However, our patient cohort was too small for such an event evaluation according to the post-hoc sample size analysis.

### Future perspective

The present study investigated the ability of a novel functional measure using CMR to predict outcome after CRT. Regional contributions to SV with this method were not sufficient in their prognostic ability, despite showing decreased longitudinal and septal function with a relatively increased lateral contribution function when compared with healthy volunteers. Currently, there is no single predictor of response to CRT, reflecting a complex syndrome with multifactorial causes. Recently a multicenter study showed that integrating multiple variables such as wasted work from echocardiography strain in combination with septal viability from CMR had the best capability to identify CRT responders [43]. Therefore, future strategies may move from simpler measures of function such as EF and myocardial dyssynchrony to more advanced combination of functional and structural measures. Which image modalities that will provide the aforementioned information remain to be determined in prospective randomized trials, and whether CMR can provide the same information as echocardiography remains to be shown.

### Limitations

CMR exams at follow-up would have been preferred to echocardiography, but CMR-compatible devices were not clinically available at the course of the study. However, all echocardiographic exams were performed by one sonographer (> 15 years of experience) at both inclusion and at follow-up, and all analyses were performed by one expert reader (> 25 years of experience) at a tertiary high-volume center.

The study group consisted of a heterogenous HF population: 49% of study participants had ischemic etiology, a group that has been shown to have a higher non-response rate to CRT than patients with non-ischemic heart failure [33, 44]. Subgroup analysis comparing ischemic HF to non-ischemic HF in this study did not show any difference in reverse remodeling between the two etiologies. Furthermore, SV_long%_, SV_lat%_, and SV_sept%_ were affected similarly in ischemic and non-ischemic HF with a trend towards lower SV_long%_ and higher SV_lat%_ in ischemic etiology, albeit not statistically significant.

The CMR protocol in the present study used LGE for tissue characterization. Since the conceptualization of the present study, T1 mapping and extra cellular volume index (ECV) measurements have become a standard part of heart failure evaluation with CMR, providing added prognostic value in non-ischemic heart failure [45, 46]. In the context of CRT selection, focal scar burden using LGE is associated with worse outcome, but diffuse scar burden using T1 mapping do not independently predict CRT outcome [47].

The post-hoc sample size analysis resulted in larger sample sizes than in the present study. Assuming there are true differences between responders and non-responders, they are probably small and would not be of clinical relevance to facilitate the selection of CRT responders on an individual level.

## Conclusions

Quantitative CMR assessment shows that patients eligible for CRT have decreased longitudinal function and that only half of the patients have a net shift of the septum towards the right ventricle during systole despite the majority of patients had LBBB. However, responders with reverse LV remodeling do not differ from non-responders regarding the longitudinal, lateral, nor septal contribution to SV. Patients with ischemic HF did not differ in regional contributions to SV compared to patients with non-ischemic HF. Thus, these regional contributions to SV are not applicable as predictors of LV remodeling in patients with LBBB eligible for CRT.

## Data Availability

The datasets generated during and/or analyzed during the current study are available from the corresponding author on reasonable request.

## Abbreviations

AVPD: Atrioventricular plane displacement
CMR: Cardiac magnetic resonance imaging
CRT: Cardiac resynchronization therapy
EDV: End-diastolic volume
EF: Ejection fraction
ESV: End-systolic volume
HF: Heart failure
LGE: Late gadolinium enhancement
LV: Left ventricle
NYHA: New York Heart Association
RV: Right ventricle
SV: Stroke volume
SV_lat%_: Lateral contribution to stroke volume
SV_long%_: Longitudinal contribution to stroke volume
SV_sept%_: Septal contribution to stroke volume
